# Posttraumatic growth-oriented peer-based training among U.S. veterans: evaluation of post-intervention and long-term follow-up outcomes

**DOI:** 10.3389/fpsyg.2023.1322837

**Published:** 2024-01-05

**Authors:** Joshua R. Rhodes, Richard G. Tedeschi, Bret A. Moore, Cameron T. Alldredge, Gary R. Elkins

**Affiliations:** ^1^Department of Psychology, Abilene Christian University, Abilene, TX, United States; ^2^Boulder Crest Institute for Posttraumatic Growth, Bluemont, VA, United States; ^3^Department of Psychology and Neuroscience, Baylor University, Waco, TX, United States

**Keywords:** veterans, posttraumatic growth, trauma, posttraumatic stress disorder, Boulder Crest Foundation

## Abstract

**Introduction:**

Exposure to trauma among U.S. military veterans occurs at a high rate, often resulting in continued difficulty with emotional adjustment and a diagnosis of posttraumatic stress disorder (PTSD). The present study provides data from 184 U.S. military veterans who completed a manualized posttraumatic-growth oriented training program during an integrative seven-day retreat.

**Methods:**

Data was collected at baseline, after program completion, and at 18-month follow-up.

**Results:**

Results on primary outcomes indicated significant increases, with medium to large effect sizes, in growth related outcomes. Specifically, there was a significant increase in scores by 54% on the posttraumatic growth outcome measure (PTGI-X) from baseline (M = 50.2, SD = 31.1) to endpoint (M = 77.4, SD = 29.6), *t*(183) = −8.78, *p* < 0.001. Also, results indicate that immediately following training (Day 7), participants reported a significant decrease of 49% on the PCL-5 from baseline (M = 39.7, SD = 17.6) to endpoint (M = 20.1, SD = 13.2), *t*(183) = 11.75, *p* < 0.001. Depression subscale scores decreased by 60% from baseline (M = 8.0, SD = 5.2) to endpoint (M = 3.2, SD = 3.0), t(183) = 10.68, *p* < 0.001; Anxiety scores decreased by 28% from baseline (M = 5.8, SD = 4.3) to endpoint (M = 4.2, SD = 3.5), *t*(183) = 4.08, *p* < 0.001; and Stress scores decreased by 50% from baseline (M = 10.0, SD = 4.4) to endpoint (M = 5.0, SD = 3.3), *t*(183) = 12.21, p < 0.001. Eighteen-month follow-up data was available for 74 participants and indicated that all significant changes in growth-related outcomes were maintained. Further, all significant changes in symptomatology-related outcomes were also maintained at follow-up.

**Discussion:**

These findings demonstrate both the immediate and the long-lasting impact of an integrative posttraumatic growth-oriented training program on psychological growth and PTSD symptom reduction among U.S. military veterans.

## Introduction

Exposure to trauma among military veterans is very prevalent, with approximately 87% of U.S. veterans reporting exposure to at least one potentially traumatic event (Wisco et al., 2014). Whether or not these reported traumatic events are directly or indirectly related to one’s military service, evidence shows the effects of trauma are causing veterans to experience serious difficulties with emotional adjustment (McKinney et al., 2017), resulting in 17.2 Veteran suicides per day in 2019, a number that has remained practically unchanged since 2001 (U.S. Department of Veterans Affairs, 2021). Of this population, individuals ages 55–74 were at the highest risk, accounting for approximately 39% of veteran deaths by suicide in 2019. A notable diagnosis aiming to capture much of this struggle with emotional adjustment following trauma is posttraumatic stress disorder (PTSD).

Presentation of PTSD related symptoms may vary across individuals from distinct wars, cultures, and genders as they have been exposed to varying specific traumas (Dutra et al., 2019). Exposure to specific traumas results in differing duration of symptoms, perception of treatment, and treatment response. For example, Chard et al. (2010) found that veterans from the Vietnam war exhibited significantly less symptom reduction following cognitive processing therapy (CPT) when compared to veterans from Operation Enduring Freedom (OEF) and Operation Iraqi Freedom (OIF). The experience of trauma related symptoms is also moderated by gender as men are more likely to report trauma resulting from exposure to combat when compared to women (Macera et al., 2014; Polusny et al., 2014) and women are more likely to experience military sexual trauma (Haskell et al., 2010; Kimerling et al., 2010). The high rate of comorbidity between PTSD and other psychiatric disorders can lead to difficulty in both its diagnoses and the identification of an optimal treatment plan. For example, while much of the current research focuses on PTSD diagnoses, many veterans also struggle with depression and anxiety. Studies estimate that 9.6% of veterans struggle with and receive a diagnosis of depression (Liu et al., 2019) and the prevalence of diagnosed anxiety disorder incidence rates varies widely from 0.01 to 23.7 per 1,000 service members (Russell et al., 2022). Differential diagnoses will highlight different aspects of PTSD symptom presentation and require different treatment modalities to avoid being ineffective or exacerbating the comorbid symptoms.

The U.S. Veterans Affairs/Department of Defense (2017) clinical practice guidelines recommend a tiered approach to PTSD treatment. The first recommendation cited to have the strongest evidence is individual, manualized, trauma-focused psychotherapy. These first-line therapies include Prolonged Exposure therapy (PE; Foa et al., 2005, 2018), Cognitive Processing Therapy (CPT; Resick et al., 2002), and Eye Movement Desensitization and Reprocessing therapy (EMDR; Shapiro, 1989; Rothbaum et al., 2005; Valiente-Gómez et al., 2017). Second tier treatment, per the VA/DOD guidelines (2017) are non-trauma, manualized therapies or pharmacotherapy. Non-trauma focused, manualized therapies include stress inoculation training and present-centered therapy. The recommended pharmacotherapies include three selective serotonin reuptake inhibitors (SSRIs; paroxetine, sertraline, and fluoxetine) and one selective norepinephrine reuptake inhibitor (SNRI; venlafaxine).

The current psychotherapeutic treatment modalities have seemed to exhibit particularly high efficacy in their reduction of PTSD symptomatology specifically. Prolonged Exposure therapy has been shown to reduce PTSD symptomatology with a pre-post effect size of *d* = 0.87 (Eftekhari et al., 2013). A meta-analysis of Cognitive Processing therapy for PTSD symptom reduction found an effect size of *g* = 1.24 when compared to control conditions (Asmundson et al., 2019). Further evidence with a more broad view is found in a meta-analysis of exposure therapies (not treatment specific), reporting a significant reduction in PTSD symptoms when compared to control conditions with an effect size of *g* = 0.86 (McLean et al., 2022). A meta-analysis examining the use of EMDR for PTSD symptom reduction found an overall effect size of *g* = −0.64 (Chen et al., 2014). The effect sizes found for the reduction of PTSD symptoms through pharmacotherapy are generally much lower, with a meta-analysis finding SSRIs to be statistically superior to placebo administration but with a small effect size (*d* = 0.23; Hoskins et al., 2015). While the evidence indicates that individuals can benefit from trauma-focused, manualized therapies, the consensus on whether they significantly outperform non-trauma-focused therapies has been called into question. Recent studies have exhibited mixed-results as to whether patients are receiving clinically meaningful benefit from first-line therapies such as PE and CPT (Steenkamp et al., 2020). Furthermore, these first-line therapies have been found to be only marginally superior to non-trauma focused therapies and active control groups (Steenkamp et al., 2015). While these first-line therapies provide great benefit for some, they are not without their limitations, namely high nonresponse, underresponse, and dropout rates among participants (Steenkamp et al., 2020). Together these findings indicate that the highly complex nature of PTSD and the management of these symptoms within a military population may not be a good match for a one-size-fits-all treatment approach (Steenkamp et al., 2020).

The framework of clinical work with military veterans has largely been focused on the identification of PTSD symptoms and symptom reduction following trauma exposure. The identification of the individual’s struggle and the goal of reducing their suffering are not only admirable, but are essential for providing help to countless veterans. The current model of PTSD and its treatment has proven to be efficacious in the reduction of PTSD specific symptoms but seems to fall short in addressing the existential needs and issues veterans face. The difficulty engaging veterans in long-term treatment across months has led to decreased satisfaction across treatment modalities, even though their completion seems to promise significant symptom reduction (Kehle-Forbes et al., 2016; Smith et al., 2019). Alternatively, intensive treatment programs (one or two weeks) have been examined as a potential way to improve on the high dropout rate and limited engagement among individuals (Hendriks et al., 2018; Watkins et al., 2018). While the evidence indicates that massed, trauma-focused therapies are beneficial (Hendriks et al., 2018), they do not directly address the potential for posttraumatic growth and there is limited research on intensive treatment programs influenced by posttraumatic growth theory.

Posttraumatic growth (PTG) is defined as positive psychological changes that can be experienced as a result of the struggle in the aftermath of traumatic or highly challenging circumstances (Tedeschi et al., 2018). Posttraumatic growth theory, largely influenced by Janoff-Bulman (1989), assumes that trauma involves emotional distress as a result of core beliefs being shattered following traumatic events. The disruption of the core belief system leads to struggle and the potential for transformative outcomes and growth. The concept of struggle refers to the difficulties encountered in the process of reconstructing one’s core beliefs. The struggle and distress can often prompt deliberative rumination within the individual. When paired with disclosure to a trusted individual, this deliberative rumination can aid in the process of reconceptualizing one’s core belief system (Tedeschi and Moore, 2021).

Posttraumatic growth has been shown to exhibit itself in five domains across individuals (Tedeschi and Calhoun, 1996; Tedeschi et al., 2017). This five-factor model has been established through factor analysis of the posttraumatic growth inventory (PTGI; Tedeschi and Calhoun, 1996; Tedeschi et al., 2017) and several other studies (Linley et al., 2007; Taku et al., 2008; Brunet et al., 2010; Lee et al., 2010). The five domains identified in prior research include: Relating to Others, Personal Strength, Appreciation of Life, New Possibilities, and Spiritual-Existential Change.

The domain of Relating to Others in PTG reflects a deeper emotional quality to relationships, often paired with an increased sense of mutual respect, disclosure, openness, and compassion (Moore et al., 2021). As trauma survivors disclose their experiences, an empathic listener can be very important in the process (Tedeschi and Calhoun, 2006).

The domain of Personal Strength is often exhibited after the individual has simply managed to survive the trauma and its aftermath. Reflection on the experience can instill a greater sense of personal strength as they recognize the self-reliance and courage it took to reach where they are (Tedeschi and Calhoun, 1996; Moore et al., 2021).

Appreciation of Life includes a new perspective that allows the individual to experience a new sense of gratitude for things previously overlooked. This increased sense of appreciation is often the result of actual loss or having narrowly escaped the loss (Tedeschi and Calhoun, 1996; Moore et al., 2021).

Traumatic experiences involve loss of things such as capabilities, roles, relationships and alter an anticipated future. In the struggle to deal with such significant loss, it is possible that new ways to live are discovered. The recognition of new possibilities for a positive future may be essential to developing PTG (Roepke and Seligman, 2015). The search for fulfillment in areas previously unconsidered, is referred to as the domain of New Possibilities (Tedeschi and Calhoun, 1996; Moore et al., 2021).

The domain of Spiritual-Existential Change may occur when individuals reconsider existential issues and potentially reconfigure their beliefs and belief systems as a result of their experience with trauma (Tedeschi and Calhoun, 1996; Moore et al., 2021). The impact of traumatic events can cause survivors to consider existential questions such as life meaning and purpose (Tedeschi and Riffle, 2016). Also, for many survivors of trauma, considerations of forgiveness, spirituality, and religious beliefs may be an important component of PTG (Schultz et al., 2010).

Rather than being a new form of therapy, PTG-based intervention is an *integrative approach* that utilizes elements and research knowledge drawn from several existing approaches, specifically: cognitive-behavioral, narrative, existential, and interpersonal. PTG-based intervention acknowledges the evolving evidence base for trauma interventions within a philosophy that proposes that trauma survivors can both achieve symptom reduction and experience transformative posttraumatic growth (Tedeschi and Moore, 2021) in the five domains of PTG. It is not simply focused on symptom reduction (although PTG-based intervention may result in reduced trauma related symptoms), but promotes and emphasizes the importance of managing emotional distress and moving toward growth that would not have been likely if not for the struggle with the traumatic events. The PTG model of intervention is unique in the emphasis on PTG, but also integrates four primary elements of existing approaches listed below.

Cognitive-behavioral interventions identify change in core beliefs and cognitions as underlying mechanisms of change. Core beliefs may be formulated in early life and people often make assumptions about themselves and the world that may go unexamined until encountering trauma that disrupts one’s “assumptive world” (Janoff-Bulman, 1989). Therefore, the PTG intervention model is directed toward schema change (Janoff-Bulman, 2006) using a variety of methods. The process of reconstructing one’s core belief system is an evidence-based approach that is a foundation for cognitive processing therapy (Resick et al., 2008) as well as PTG-based intervention. Further, many trauma survivors must achieve a sufficient degree of emotional regulation before being able to tolerate the emotional stress associated with schema change. Therefore, psychoeducation about trauma reactions and teaching emotional regulation methods (i.e., relaxation, mindfulness, present-moment awareness, etc.) are an integral component of the PTG integrative intervention approach.

PTG-based intervention also integrates the concept of developing a new life narrative into it’s approach. With the revision of core beliefs, trauma survivors are often tasked with making personal decisions about what kind of life they wish to have in the future and incorporating the past trauma into their personal narrative of their past, present, and future. Narrative therapies, such as those delivered through expressive writing and reflection have been shown to have positive effects on PTG (Hijazi et al., 2014). Interventions that integrate narrative development have been shown to be of benefit with a range of trauma survivors (Neimeyer, 2006; Smyth et al., 2008).

Along with core belief and narrative examination, the struggle with the aftermath of trauma often leads to an awareness of existential questions such as meaning and purpose of trauma and their lives. Survivors may struggle with questions about fairness, justice, and finding new meaning and purpose following significant loss (Frankl, 1962). The existential element is drawn from the concepts within logotherapy and existential therapy generally (Tedeschi and Riffle, 2016). Examination of existential issues is deliberately addressed within the PTG-based approach.

Interpersonal and “common factors” have been shown to be significant components of most psychotherapeutic interventions (Norcross and Wampold, 2011). In the PTG-based intervention, individuals called “expert guides” are individuals trained in providing a supportive environment, non-judgmental listening, unconditional positive regard, and activities toward promoting growth beyond symptom reduction (Calhoun and Tedeschi, 2013). This interpersonal element provides the foundation for trauma survivors to expand their support system, construct new core beliefs, and personal narrative that promotes posttraumatic growth.

PTG-based intervention is provided by individuals trained in providing “expert companionship” often referred to as “expert guides.” PTG-based intervention is conceptualized as a training program that follows the natural process following the aftermath of trauma. The goals are not limited to symptom reduction, but include managing distress, and achieving growth in multiple domains. There are five general components in the structure of PTG-based intervention (Calhoun and Tedeschi, 2013).

Psychoeducation is provided regarding how trauma symptoms develop (Barlow, 2014) and understanding distressing symptoms (Meichenbaum, 2012). Further, psychoeducation is provided regarding the potential of PTG, the domains of PTG, and sharing personal examples. This includes a discussion of how core beliefs have been disrupted and how a reconceptualization and development of new core beliefs can be transformative toward living well and thriving in the aftermath of trauma (Calhoun and Tedeschi, 2013; Tedeschi and Moore, 2021). In addition, assurance about facing and addressing existential questions is provided within a caring and non-judgmental interpersonal relationship.

Teaching and practicing methods of emotional regulation is integrated throughout the structure of PTG-based intervention. This includes relaxation, focus on breathing, grounding techniques, mindfulness exercises, meditation, relaxed music listening, and exercise. Experiential practice is demonstrated and emphasized over intellectual understanding of these emotional regulation techniques. It is beneficial to allow survivors to experience a variety of ways to regulate emotions and select personal preferences to apply to their own lives (Cooper et al., 2019).

Disclosure is essential within the PTG-based intervention structure. Disclosure is modeled by the “expert guides” and a safe and supportive environment is fostered. However, in contrast to exposure-based therapies (Peterson et al., 2019), the disclosure is not focused on the specifics of the traumatic events, but on the impact of these events on the individual’s core belief system about self, others, the world, and the future (Williams et al., 2019). Disclosure about one’s personal life story broadly defined is encouraged within a non-judgmental context. Openness to sharing key personal life events, influences, successes, perceived failures, and decreasing defensiveness are important aspects of self-reflection and a sense of being accepted.

Development of a personal “life story” brings together an understanding of key past experiences, perhaps extending into childhood. It is not simply disclosure about the traumatic event. Rather, the development of the survivor’s personal story includes the trauma in the context of entire past and future. It includes looking forward and consideration of new possibilities for the future and new ways of understanding the past. The narrative may bring up past events, regrets, guilt, or unresolved anger. Some aspects may require acceptance and other change in moving toward the future with growth. Development of a new “life story” narrative is achieved with the support of the “expert guide” and in the process of disclosure.

As posttraumatic growth occurs, survivors may have new insights, goals, sense of meaning and purpose. An awareness of ways they can support the growth of others who may have experienced their own traumas, leads to a stronger connection with family and community. This can be manifested in new goals in life and awareness of service to help others and sharing their experience of PTG.

The Warrior PATHH (Progressive and Alternative Training for Helping Heroes) program is the flagship program of the Boulder Crest Foundation (BCF), a non-profit organization focused on the psychological health of U.S. veterans. The Warrior PATHH program is a 7 days intensive residency program developed to provide PTG-based training and experiences to veterans. The program consists of 48 psychoeducational modules, which are described in a 200-page guide developed for program instructors. Although the program does not offer traditional, evidenced-based psychotherapies, the program does utilize a variety of complementary and alternative interventions (e.g., mindfulness/meditation, yoga, equine therapy) and traditional psychotherapeutic techniques (e.g., psychoeducation, distress management, relationship building, narrative development, goal setting). A unique aspect of Warrior PATHH is that it is peer-delivered and is not run or managed by mental health professionals. This is an important component of the program as those working within Warrior PATHH are veterans who understand the unique needs and professional culture of those who attend the program. The peers who deliver the bulk of the program are combat veterans who have undergone several months of training from peer leaders with years of experience delivering the program as well as licensed mental health professionals. Peers delivering the program receive ongoing training and consultation from these same peer leaders and professionals. Consequently, Warrior PATHH is considered a training program as opposed to a treatment program. Following the residential portion of the program, a structured 18 months of follow-up is offered through a web-based series of meetings and assignments. A more complete description of the Warrior PATHH program can be found in Moore et al. (2021).

While the PTG-based intervention approach of Warrior PATHH has been well developed and manualized, to date there has been limited outcome research. A small pilot study of the Warrior PATHH posttraumatic growth-based intervention program found significant, large reductions in symptomatology including PTSD, insomnia, and negative affect (Moore et al., 2021). Additionally, results indicated significant increases in areas of PTG and psychological flexibility. While these results are encouraging, they do not report immediate outcomes of study participants, resulting in the inability to gather a full picture of immediate and long-term effects. Without the report of immediate outcomes, one does not know about the trajectory of benefit to participants and cannot determine any lasting changes. The purpose of the present study was to address this gap in research by conducting a retrospective evaluation of a much larger sample of veterans with PTSD symptoms who completed the Warrior PATHH program. Outcome data were collected at baseline, after the 7 day program was completed, and at 18 months follow-up.

## Method

Participants were United States combat Veterans completing the Warrior PATHH program. Participants were self-referred and most commonly learned about the program from other veterans and family members familiar with the training program. Participation in the training program was free of cost to all participants and they received no compensation for completing the training. Inclusion criteria for the Warrior PATHH program were individuals who were (1) U.S. military Veterans and (2) had a previous history of trauma. Individuals were excluded from participation if they (1) were diagnosed with any disorder that might require hospitalization, such as psychosis, substance abuse, or active suicidality.

Data collection for all participants occurred prior to the initiation of the program (Day 0), which will be referred to as *baseline*; at the end of the training (Day 7), which will be referred to as *endpoint*; and 18 months following the completion of training, which will be referred to as *follow-up*. The formal evaluation at baseline, endpoint, and follow-up was completed through the administration of an electronic questionnaire. A selection of measurements relating to growth and symptomatology domains are reported in this manuscript. All measurement tools listed were collected at baseline, endpoint, and 18-month follow-up.

### Measures

Posttraumatic Growth Inventory – Expanded (PTGI-X). The PTGI-X (Tedeschi et al., 2017) is a 25-item self-report measure used to assess the extent to which individuals report positive psychological change following the experience of a traumatic event. Five subscales of this measure assess changes in one’s perception of new possibilities, relating to others, personal strength, appreciation of life, and spiritual-existential change. Individual items are on a 6-point Likert scale ranging from “I did not experience this change” to “I experienced this change to a very great degree.” Good internal consistency (*α* = 0.90; Tedeschi et al., 2017) and content validity has been shown in research (Shakespeare-Finch et al., 2013).

**Positive and Negative Affect Schedule (PANAS)**. The PANAS (Watson et al., 1988) is a 20-item self-report measure for both positive and negative affect. Individual item responses are on a 5-point frequency scale ranging from “not at all” to “extremely.” Strong reliability for both the positive (*α* = 0.89) and negative subscale (*α* = 0.85) and construct validity has been reported in addition to substantial available normative data (Crawford and Henry, 2004).

**Integration of Stressful Life Experiences Scale (ISLES)**. The ISLES (Holland et al., 2010) is a 16-item self-report measure used to assess the extent of meaning made following a stressful life experience. Individual items are on a 5-point Likert scale ranging from “strongly agree” to “strongly disagree.” The ISLES has exhibited strong internal consistency, strong convergent validity, and moderate test–retest reliability (Holland et al., 2010).

**Posttraumatic Stress Disorder Checklist DSM 5 (PCL-5)**. The PCL-5 (Weathers et al., 2013) is a 20-item self-report measure assessing DSM-5 symptoms of PTSD. Individual item responses are on a 5-point frequency scale ranging from “not at all” to “extremely.” Strong construct validity (*α* = 0.92) and test–retest reliability (*r* = 0.57) has been found in veteran samples (Bovin et al., 2015; Dutra et al., 2019).

**Depression, Anxiety, and Stress Scale (DASS)**. The DASS (Antony et al., 1998) is a 21-item self-report measures of the presence and degree of depression, anxiety, and stress-related symptoms. Individual item responses are on a 4-point frequency scale ranging from “never” to “almost always.” Adequate test–retest reliability (*α* = 0.86–0.90; Gloster et al., 2008) in addition to discriminate and convergent validity has been shown in clinical samples (Brown et al., 1997).

**Insomnia Severity Index (ISI)**. The ISI (Bastien et al., 2001) is a 7-item self-report measure, based on DSM-IV and the International Classification of Sleep Disorders criteria, used to assess insomnia over the past 2 weeks. Individual items are assessed on a 5-point Likert scale ranging from “none” to “very severe.” High reliability and validity have been shown for the ISI in both clinical (*α* = 0.91) and community (*α* = 0.90) samples (Morin et al., 2011).

### Intervention

Framed as an intensive training program for veterans, the evaluated program is the Warrior PATHH (Progressive and Alternative Training for Helping Heroes). The manualized, 7 days training program is based on posttraumatic growth theory and its intervention model (Tedeschi and McNally, 2011; Calhoun and Tedeschi, 2013; Tedeschi and Moore, 2016, 2018). During this 7 days period, participants are immersed in an all-day intensive regimen combining education and experiential activities. Participants who attend the Warrior PATHH program experienced the following five elements in accordance with the PTG model: psychoeducation about physiological and psychological trauma response and psychological growth; emotion regulation training, including mindfulness and meditative techniques; constructive self-disclosure about trauma and its aftermath that occurs naturally through casual discourse; non-trauma focused narrative development integrating perspectives of past, present, and future; and service goals that are developed to carry out the lessons learned about the value of life, living courageously, and understanding those who have not had the same experiences (Moore et al., 2021). Representing a relational approach to the intervention called Expert Companionship, those providing the intervention are referred to as “expert guides.” All expert guides that were responsible for delivering the intervention were U.S. combat veterans who underwent several months of training with established expert guides and mental health professionals trained in the PTG-based intervention. Delivered as a peer-to-peer training program, the Warrior PATHH program took place during a 7 days period at the BCF facility in Bluemont, Virgina, between February 2019 and December 2021.

### Analyses

Descriptive statistics at baseline, endpoint, and follow-up were calculated for each of the outcome measures. To determine immediate post-intervention effects, paired samples *t*-tests were conducted for each outcome. Paired samples *t*-tests are used to determine the statistical significance of the difference in scores on outcome measures from baseline to endpoint. The independent variable in these analyses was study time point which had two levels, baseline (Day 1) and endpoint (Day 7). Dependent variables in these analyses were growth domains, including posttraumatic growth, positive affect, and integration of stressful life experiences; and symptomatology domains, including PTSD symptoms, depression, anxiety, stress, negative affect, and insomnia.

To determine the lasting effects of the Warrior PATHH program on growth and symptomatology domains a series of repeated measures analysis of variance (repeated measures ANOVA) were conducted, which are extensions of the paired-samples *t*-tests. The independent variable for these repeated measures ANOVAs was “time” (baseline, endpoint, and follow-up). The dependent variables for these repeated measures ANOVAs were the specific outcome measures. In the event of a significant main effect of time, pairwise comparisons using a Bonferroni correction were examined to determine differences between specific study time points.

To determine the moderating effect of gender on immediate and lasting post-intervention effects on growth and symptomatology domains, multiple analyses of covariance (ANCOVA) were conducted. The independent variable for these ANCOVAs examining immediate effects was “gender,” the dependent variables were the scores on the specific outcome measures at endpoint, and the covariates were the baseline score of the specific outcome. The independent variable for the ANCOVAs examining lasting effects was “gender,” the dependent variables were the scores of the specific outcome measure at follow-up, and the covariates were the endpoint score of the specific outcome.

## Results

Participants were 184 United States combat veterans (male = 155, female = 29) who completed the Warrior PATHH training program between February 2019 and December 2021. The most common U.S. military branch of service represented was Army (*n* = 110), with the second most frequent being Marine Corps. (*n* = 32). Frequencies of all participant demographic variables can be found in Table 1.

**Table 1:** Participant demographics.

Demographic variable	Frequency
Gender	
Male	155
Female	29
Age range	
23–27	3
28–32	15
33–37	46
38–42	43
43–47	24
48 or older	53
Branch of service	
Air Force	18
Army	110
First Responder	12
Marine Corps	32
Navy	12
Military Rank	
E1–E4	32
E5–E6	69
E7 or above	41
Officer/Warrant Officer	31
Not specified	11

All participants (*n* = 184) provided complete data at baseline and endpoint and were included in all analyses examining post-intervention changes. At the optional 18 months follow-up time point, 74 individuals completed outcome measures. Due to the nature of the statistical analyses examining long-term changes, only the 74 individuals who provided data at all three time points were included in the analyses of long-term changes at 18 months.

### Post-intervention changes in growth domains

A paired samples *t*-test was used to compare scores on each growth-related outcome variable between baseline and endpoint. Results indicate that immediately following training (Day 7), participants reported a significant increase in scores by 54% on the posttraumatic growth outcome measure (PTGI-X) from baseline (M = 50.2, SD = 31.1) to endpoint (M = 77.4, SD = 29.6), *t*(183) = −8.78, *p* < 0.001. Additionally, there was a significant increase in scores by 45% on the positive affect subscale of the PANAS from baseline (M = 25.8, SD = 8.4) to endpoint (M = 37.5, SD = 6.9), *t*(183) = −14.84, *p* < 0.001. Finally, there was a significant increase in scores by 29% on the ISLES from baseline (M = 39.2, SD = 12.9) to endpoint (M = 50.4, SD = 10.9), *t*(183) = −9.13, *p* < 0.001. Table 2 contains calculated effect sizes for each paired sample comparison.

**Table 2:** Growth domains effect sizes.

Outcome	Effect Size (*d*)
PTG	−0.65
Positive affect	−1.09
ISLES	−0.67

Results from paired samples *t*-tests for scores on each subscale of the PTGI-X indicate a significant difference between baseline and endpoint for New Possibilities (M = 10.6, SD = 7.3, M = 15.7, SD = 6.3, respectively), *t*(183) = −7.13, *p* < 0.001; Personal Strength (M = 8.9, SD = 5.9, M = 13.8, SD = 4.8, respectively), *t*(183) = −9.01, *p* < 0.001; Appreciation of Life (M = 8.22, SD = 4.2, M = 9.7, SD = 3.9, respectively), *t*(183) = −3.71, *p* < 0.001; Relating to Others (M = 12.6, SD = 9.6, M = 21.5, SD = 9.5, respectively), *t*(183) = −9.47, *p* < 0.001; and Spiritual-Existential Change (M = 10.0, SD = 8.4, M = 16.6, SD = 8.5, respectively), *t*(183) = −7.54, *p* < 0.001. Medium effect sizes were found for scores on the subscales of Relating to Others (Cohen’s *d* = −0.70), Personal Strength (Cohen’s *d* = −0.66), Spiritual-Existential Change (Cohen’s *d* = −0.56), and New Possibilities (Cohen’s *d* = −0.53), and there was a small effect size for scores on Appreciation of Life subscale (Cohen’s *d* = −0.27).

### Post-intervention changes in symptomatology domains

Next, a paired samples *t*-test was used to compare scores on each symptomatology-related outcome variable between baseline and endpoint. Results indicate that immediately following training (Day 7), participants reported a significant decrease in scores by 49% on the PCL-5 from baseline (M = 39.7, SD = 17.6) to endpoint (M = 20.1, SD = 13.2), *t*(183) = 11.75, *p* < 0.001. A significant decrease in scores was reported for all subscales of the DASS. Depression subscale scores decreased by 60% from baseline (M = 8.0, SD = 5.2) to endpoint (M = 3.2, SD = 3.0), *t*(183) = 10.68, *p* < 0.001; anxiety subscale scores decreased by 28% from baseline (M = 5.8, SD = 4.3) to endpoint (M = 4.2, SD = 3.5), *t*(183) = 4.08, *p* < 0.001; and stress subscale scores decreased by 50% from baseline (M = 10.0, SD = 4.4) to endpoint (M = 5.0, SD = 3.3), *t*(183) = 12.21, *p* < 0.001. Participant scores on the ISI significantly decreased by 36% from baseline (M = 14.3, SD = 6.9) to endpoint (M = 9.1, SD = 5.3), *t*(183) = 8.03, *p* < 0.001. Finally, scores on the negative affect subscale of the PANAS significantly decreased by 16% from baseline (M = 25.4, SD = 8.2) to endpoint (M = 21.4, SD = 7.4), *t*(183) = 4.89, *p* < 0.001. Table 3 contains calculated effect sizes for each paired sample comparison.

**Table 3:** Symptomatology domains effect sizes.

Outcome	Effect Size (*d*)
PTSD	0.87
Depression	0.79
Anxiety	0.30
Stress	0.90
Insomnia	0.59
Negative affect	0.36

### Long term changes in growth domains

A series of independent samples *t*-tests were first conducted to determine if the 74 participants who completed follow-up measures were representative of the entire sample. The test variable for each of these analyses was the baseline score of each outcome measure. The grouping variable used was (A = Participants who completed baseline and endpoint data; *n* = 110) and (B = participants who completed follow-up measures; *n* = 74). Results of these *t*-tests indicate that there were no significant differences between groups on any outcome measure at baseline, with the exception of baseline depression (Mean Difference = 1.59, *p* = 0.041) and baseline ISLES (Mean Difference = 4.32, *p* = 0.023) scores. Overall, these findings suggest a follow-up sample representative of the entire sample.

A series of repeated measures ANOVAs were then conducted to examine changes in growth-related outcome measures over three time points (baseline, endpoint, and follow-up). The analysis on PTGI-X scores revealed a significant main effect of time, *F*(2,146) = 21.26, *p* < 0.001. Pairwise comparisons indicate that PTGI-X scores significantly increased by 27.1 points (52%) from baseline to endpoint (*p* < 0.001) and remained stable until follow-up, exhibited by a further increase of 0.85 points (*p* = 1.00) and a total increase of 54% between baseline and follow-up. The analysis on positive affect subscale scores also revealed a significant main effect of time, *F*(2,146) = 56.09, *p* = <0.001. Pairwise comparisons indicate that scores on the positive affect subscale significantly increased by 11.1 points (41%) from baseline to endpoint (*p* < 0.001). Results indicate that between endpoint and follow-up, positive affect subscale scores significantly decreased by 8.2 points (*p* < 0.001); however, there remained a significant difference between baseline and follow-up scores (*p* = 0.010) exhibited by a total increase of 11% between baseline and follow-up. Regarding the analysis on ISLES scores, Mauchly’s test indicated that the assumption of sphericity had been violated, *χ*^2^ (2) = 6.70, *p* = 0.035, therefore the degrees of freedom were corrected using Greenhouse-Geisser estimates of sphericity (*ε* = 0.918). The results indicate a significant main effect of time, *F*(1.84, 134.09) = 23.99, *p* < 0.001. Pairwise comparisons indicate that ISLES scores significantly increased by 9.69 points (23%) from baseline to endpoint (*p* < 0.001) and remained stable until follow-up, exhibited by a non-significant increase of 1.42 points (*p* = 1.00) and a total increase of 27% from baseline to follow-up. Table 4 includes means and standard deviations for each growth-related outcome measure at each time point.

**Table 4:** Long-term changes in growth domains.

Outcome	Baseline		Endpoint		Follow-up	
	Mean	SD	Mean	SD	Mean	SD
PTG	52.03	29.20	79.12	30.47	79.97	31.40
Positive Affect	27.20	7.94	38.26	5.74	30.09	7.98
ISLES	41.66	11.60	51.35	10.49	52.77	9.86

The analyses on PTGI-X subscale scores revealed a significant main effect of time for Appreciation of Life, *F*(2,146) = 3.25, *p* = 0.041; Personal Strength, *F*(2,146) = 24.66, *p* < 0.001; New Possibilities, *F*(2,146) = 13.91, *p* < 0.001; Relating to Others *F*(1.81, 131.96) = 24.21, *p* < 0.001; and Spiritual-Existential Change, *F*(1.75, 127.84) = 17.96, *p* < 0.001. Mauchly’s test indicated that the assumption of sphericity had been violated for the scores on subscales Relating to Others, *χ*^2^ (2) = 8.10, *p* = 0.017, and Spiritual-Existential Change, *χ*^2^ (2) = 11.03, *p* = 0.004, therefore the degrees of freedom were corrected using Greenhouse-Geisser estimates of sphericity (*ε* = 0.904, *ε* = 0.876; respectively). Pairwise comparisons indicate that all subscales with a significant main effect of time, with the exception of the Appreciation of Life subscale, exhibited a significant increase in scores from baseline to endpoint, followed by stability through follow-up. This stability through follow-up was exhibited by a non-significant decrease in scores for Personal Strength and New Possibilities subscales and by a non-significant increase in scores for the Relating to Others and Spiritual-Existential Change subscales. Table 5 includes means and standard deviations for each PTGI-X subscale at each time point.

**Table 5:** Long-term changes in PTGI-X subscales.

Outcome	Baseline		Endpoint		Follow-up	
	Mean	SD	Mean	SD	Mean	SD
Appreciation of Life	8.35	4.20	9.89	3.87	9.69	4.04
Personal Strength	8.76	5.49	13.95	5.00	13.65	5.30
New Possibilities	10.73	7.60	16.00	6.46	15.93	6.87
Relating to Others	13.62	8.78	22.34	9.37	22.68	9.28
Spiritual-Existential Change	10.57	8.42	16.95	8.89	18.03	8.43

### Long term changes in symptomatology domains

A series of repeated measures ANOVAs were conducted to examine changes in symptomatology-related outcome measures over three time points (baseline, endpoint, and follow-up). Mauchly’s Test of Sphericity indicated that the assumption of sphericity had been violated for PCL-5 scores, *χ*^2^ (2) = 15.82, *p* < 0.001, depression scores, *χ*^2^ (2) = 23.17, *p* < 0.001, anxiety scores, *χ*^2^ (2) = 12.80, *p* = 0.002, ISI scores, *χ*^2^ (2) = 12.91, *p* = 0.002, and negative affect scores, *χ*^2^ (2) = 11.52, *p* = 0.003, and therefore a Greenhouse-Geisser correction was used for each of these analyses. The analysis of PCL-5 scores revealed a significant main effect of time, *F*(1.67, 146) = 33.87, *p* < 0.001. Pairwise comparisons indicate that PCL-5 scores significantly decreased by 15.66 points (43%) from baseline to endpoint (*p* < 0.001) and remained stable until follow-up, exhibited by a further decrease of 2.43 points (*p* = 1.00) and a total decrease of 49% from baseline to follow-up. The analysis of depression subscale scores revealed a significant main effect of time, *F*(1.57, 114.49) = 21.05, *p* < 0.001. Pairwise comparisons indicate that depression subscale scores significantly decreased by 3.37 points (48%) from baseline to endpoint (*p* < 0.001) and remained stable until follow-up, exhibited by a further decrease of 0.31 points (*p* = 1.00) and a total decrease of 53% from baseline to follow-up. The analysis of anxiety subscale scores revealed a significant main effect of time, *F*(1.72, 125.55) = 9.09, *p* < 0.001. Pairwise comparisons indicate that anxiety subscale scores did not significantly decrease from baseline to endpoint (*p* = 0.534), but did significantly decrease by 1.55 points from endpoint to follow-up (*p* = 0.029) resulting in a total decrease of 45% from baseline to follow-up. The analysis of stress subscale scores revealed a significant main effect of time, *F*(2, 146) = 29.30, *p* < 0.001. Pairwise comparisons indicate that stress subscales scores significantly decreased by 4.22 points (44%) from baseline to endpoint (*p* < 0.001) and remained stable until follow-up exhibited by a non-significant further decrease in scores by 0.04 points (*p* = 1.00) and a total decrease of 44% from baseline to follow-up. The analysis of ISI scores revealed a significant main effect of time *F*(1.72, 125.42) = 15.60, *p* < 0.001. Pairwise comparisons indicate ISI scores significantly decreased by 4.38 points (32%) from baseline to endpoint (*p* < 0.001) and remained stable until follow-up, exhibited by a non-significant further decrease in scores by 0.32 points (*p* = 1.00) and a total decrease of 34% from baseline to follow-up. The analysis of negative affect subscale scores revealed a significant main effect of time *F*(1.74, 127.19) = 20.33, *p* < 0.001. Pairwise comparisons indicate that negative affect subscale scores did not significantly decrease from baseline to endpoint (*p* = 0.674), but did significantly decrease by 5.00 points from endpoint to follow-up (*p* < 0.001), resulting in a total decrease of 27% from baseline to follow-up. Table 6 includes means and standard deviations for each symptomatology measure at each time point.

**Table 6:** Long-term changes in symptomatology domains.

Outcome	Baseline		Endpoint		Follow-Up	
	Mean	SD	Mean	SD	Mean	SD
PTSD	36.80	18.20	21.14	13.05	18.70	14.20
Depression	6.99	5.27	3.62	3.25	3.31	3.74
Anxiety	5.55	4.47	4.62	3.89	3.07	3.35
Stress	9.68	4.59	5.46	3.33	5.42	4.09
Insomnia	13.77	7.29	9.39	5.51	9.07	5.74
Negative Affect	24.00	7.59	22.51	6.48	17.51	6.87

### Gender differences in post-intervention outcomes

Multiple one-way ANCOVAs were conducted to determine a statically significant difference between male and female participants on each growth and symptomatology-related outcome measure at endpoint, controlling for participant baseline scores. After adjustment for pre-intervention scores, there was only one outcome measure, DASS anxiety subscale scores, that resulted in a statistically significant difference in post-intervention scores between male and female participants, *F*(1, 181) = 4.17, *p* = 0.043, partial *η*^2^ = 0.023. Analysis of covariance results, in addition to adjusted means and standard errors, for all outcome measures can be found in Table 7.

**Table 7:** Effect of gender on post-intervention outcomes.

Outcome	Male (*n* = 155)		Female (*n* = 29)		*F*	*p*	partial *η*^2^
	Mean^a^	SE	Mean^a^	SE			
PTG	77.26	2.40	77.89	5.58	0.011	0.918	0.000
Positive Affect	37.47	0.56	37.51	1.29	0.001	0.976	0.000
ISLES	50.36	0.88	50.82	2.05	0.042	0.838	0.000
PTSD	20.58	1.06	17.41	2.47	1.38	0.242	0.008
Depression	3.34	0.24	2.39	0.56	2.38	0.125	0.013
Anxiety	4.45	0.28	3.00	0.65	4.17	0.043	0.023
Stress	5.19	0.26	4.00	0.61	3.22	0.074	0.017
Insomnia	9.21	0.43	8.17	1.00	0.923	0.338	0.005
Negative Affect	21.66	0.60	19.69	1.39	1.70	0.194	0.009

a Adjusted means after controlling for baseline scores are presented.

### Gender differences in long-term outcomes

Finally, multiple one-way ANCOVAs were conducted to determine a statistically significant difference between male and female participants on each growth- and symptomatology-related outcome measure at follow-up, controlling for participant endpoint scores. After adjustment for endpoint scores, no outcome measure at follow-up indicated a statistically significant difference between male and female participants. Analysis of covariance results, in addition to adjusted means and standard errors, for all outcome measures at follow-up can be found in Table 8.

**Table 8:** Effect of gender on long-term outcomes.

Outcome	Male (*n* = 63)		Female (*n* = 11)		*F*	*p*	partial *η*^2^
	Mean^a^	SE	Mean^a^	SE			
PTG	79.77	3.98	81.13	9.52	0.017	0.896	0.000
Positive Affect	29.93	1.02	31.01	2.44	0.167	0.684	0.002
ISLES	52.05	1.23	56.90	2.94	2.33	0.132	0.032
PTSD	19.56	1.79	13.80	4.28	1.54	0.219	0.021
Depression	3.51	0.47	2.17	1.14	1.19	0.279	0.017
Anxiety	3.28	0.42	1.83	1.02	1.71	0.196	0.023
Stress	5.73	0.51	3.66	1.22	2.44	0.123	0.033
Insomnia	9.21	0.73	8.23	1.75	0.267	0.607	0.004
Negative Affect	17.67	0.88	16.62	2.11	0.211	0.647	0.003

a Adjusted means after controlling for endpoint scores are presented.

## Discussion

The present study examined the impact of a manualized, posttraumatic growth-oriented training program on various growth and symptomatology-related outcome variables among U.S. military veterans. The utilized training program, the Warrior PATHH program, is not conceptualized as a psychotherapeutic intervention. Rather, this manualized training program is peer based and developed to help veterans develop the tools necessary to increase their capacity to regulate thoughts, emotions, and actions in a civilian environment. The primary focus of the Warrior PATHH training is the facilitation of PTG and its five domains. However, the integrated nature of the training means that many elements of empirically based PTSD treatments have been incorporated into the design. The incorporation of these elements implies that completion of the training program should also have an impact on the presentation of PTSD related symptomatology.

### Post-intervention outcomes

In accordance with our hypotheses regarding immediate post-intervention changes, findings revealed significant improvements on all growth-related outcome measures immediately following the training. Specifically, participants reported a 54% increase in scores on the posttraumatic growth inventory (PTGI-X) from baseline to endpoint, indicating that participants experienced a noteworthy enhancement in their perception of posttraumatic growth-related domains in the aftermath of the training. Further evidence of this growth is exhibited by significant improvements on all five subscales of the PTGI-X. Similarly, a significant increase in scores was observed for the positive affect subscale of the PANAS, exhibited by a 45% increase in scores from baseline to endpoint. This suggests that participants experienced notable improvement in their overall positive emotional states following the training. Finally, findings revealed a significant increase in scores on the ISLES from baseline to endpoint by 29%, indicating that participants are able to report an increased sense of meaning following their previous trauma exposure. The observed effect sizes for each paired sample comparison, as presented in Table 2, further support the robustness of this study’s findings. The magnitude of change found in each growth-related outcome variable provides additional evidence for the meaningful impact of training program on participants self-reported experiences. Overall, the results provide strong empirical support for the effectiveness of the Warrior PATHH training program in promoting posttraumatic growth, positive affect, and the integration of stressful experiences immediately following training.

While the program was not focused on treatment, it is very noteworthy that participants reported significant reduction of PTSD symptomatology scores by 49% from baseline to endpoint. This suggests a notable alleviation of PTSD related symptoms among participants. Additional significant reductions were found for depression (60%), anxiety (28%), and stress (50%) scores from baseline to endpoint. Finally, insomnia symptom scores decreased by 36% and negative affect by 16% from baseline to endpoint. The degree to which each of these domains changed from baseline to endpoint is demonstrated by paired sample comparisons in Table 3.

### Long term 18 months follow-up outcomes

The observed increase in growth domains and decrease in symptomatology domains across participants immediately following the Warrior PATHH program sheds light on just how powerful an intensive training of this type can be. However, it is important to examine whether the improvements are maintained long-term after the individuals return to their normal environment. For example, each individual who attends the training has removed themselves from many of the daily stressors they experience, is given a strict schedule to adhere to, and is surrounded by individuals with similar experiences who are more likely to understand their struggles. Once the individual returns home, they are again exposed to any routine daily stressors, are responsible for their own schedule, and may not have the same level of empathetic community found during training. Significant results at the 18 months follow-up would indicate a high level of impact from the training, resulting in changes that are maintained for one and a half years post-training.

Data from repeated measures ANOVAs indicates that, for those who provided data at all three time points (*n* = 74), the significant increase in all growth-related outcome measure scores from baseline was maintained at the 18 months follow-up. Both PTGI-X and ISLES scores did not differ significantly from endpoint to follow-up, indicating stability and lasting benefits from the training program. Although there was a significant decrease in positive affect scores between endpoint and follow-up assessments, the scores remained significantly higher than baseline, indicating a lasting positive impact of the training.

Scores on all symptomatology-related outcome measures that exhibited a significant decrease from baseline to endpoint remained stable at the 18 months follow-up, maintaining their significant decrease from the baseline assessment. While both anxiety and negative affect scores experienced a non-significant decrease from baseline to endpoint, scores for both domains experienced a significant decrease from baseline to follow-up assessment.

### Examination of gender of participants

The present study also aimed to investigate the potential differences between male and female participants on various growth and symptomatology-related outcome measures at both endpoint and follow-up. The results of multiple one-way ANCOVA’s indicate that after adjusting for pre-intervention scores, there was only a single outcome measure, DASS anxiety subscale scores, that showed a small but statistically significant difference in post-intervention scores between male and female participants. The effect size, as indicated by the partial *η*^2^ value, was very small, only explaining approximately 2.3% of the variance in scores on the anxiety subscale.

Interestingly, no significant gender differences were found for any other growth or symptomatology related outcome measure at endpoint or follow-up, after controlling for the appropriate covariates. These findings indicate that on almost all outcome variables assessed, male and female participants showed similar levels of improvement at endpoint and maintenance of such improvement at follow-up.

The discrepancy in samples size for the analyses examining gender differences at follow-up should be noted, as male participants (*n* = 63) far outnumbered female participants (*n* = 11). This overall small sample of participants may have influenced the statistical power to detect differences between genders. Furthermore, the smaller sample size of female participants may have limited the ability to detect any effects that could have been present.

The findings of these analyses are encouraging in that both female and male veterans seem to experience the same level of benefit from the posttraumatic growth-oriented training employed in this study. More specifically, the large increases in PTG and decreases in PTSD symptomatology were exhibited by participants, regardless of gender. These results indicate that the current training protocol seems to adequately address the needs of both male and female veterans, however future studies with larger and more balanced gender samples are needed to further explore the potential influence of gender on the assessed variables.

### PCL-5 threshold scores

The PCL-5 was used to assess prevalence and severity of PTSD symptomatology among participants completing the training program. The high impact of the training on post-intervention and long-term follow up PCL-5 scores warrants further investigation. While the use of the PCL-5 was strictly to assess prevalence and severity of PTSD symptoms, and not a tool used to make a diagnosis, an investigation of the participants who fall above and below the diagnosis threshold score can be informative. For example, a provisional PTSD diagnosis can be made with a score of 33 or greater on the PCL-5. At the baseline assessment (*n* = 184), there were 120 individuals with scores greater than 33 and who theoretically would qualify for this provisional diagnosis. Immediately following the training, only 31 individuals had scores greater than 33, indicating that 89 individuals (74%) have theoretically reduced symptomatology to a point that would no longer meets the diagnostic threshold. For those who provided data at all three study time points (*n* = 74), there were 40 individuals with PCL-5 scores greater than 33, meeting criteria for a provisional PTSD diagnosis at the baseline assessment. At the endpoint assessment, only 12 individuals had scores greater than 33, indicating 28 fewer individuals (70%) met the diagnostic criteria regarding PCL-5 scores. One and a half years later at the 18 months follow-up assessment, 15 individuals had PCL5 scores greater than 33 indicating a rise in PTSD symptoms over the course of 18 months for 3 of the individuals. This rise in the number of individuals who exceed the diagnostic threshold is expected over time but highlights the need for continued support beyond the immediate post-intervention period.

Together, these results underscore the positive impact of the Warrior PATHH training program in reducing PTSD symptomatology among veterans, despite its primary focus of increasing PTG. The substantial impact on reported PTSD symptomatology, exhibited by a 49% decrease in reported symptoms from baseline to endpoint, warrants further investigation to identify factors associated with increased PTG and symptom improvement.

### Limitations

The present study is not without its limitations, primarily the lack of a control group. While a single-group design is appropriate for the current study, future studies should consider employing randomized controlled trail designs to further investigate the effects of the training and help establish causal relationships. The ethics and complexity of adding a control group to a study of this nature should be carefully examined to account for many of the factors present in the current training protocol.

While demographic variables were collected, certain variables that may be of interest to the field were not collected in this study (i.e., nature of trauma, time since trauma, and age of service). Analysis of the relationship between these and outcome variables may identify certain patterns in training benefit.

Finally, the findings of this study were based on a specific manualized intervention within a specific population, U.S. military veterans. While the sample size and analyses allow for the results to be generalizable to U.S. military veteran populations, they may not be generalizable to other study populations. Future studies should consider adapting and employing the utilized training among other populations that are at an increased risk of trauma exposure.

## Conclusion

In conclusion, the results of this study demonstrated significant improvements in growth-related outcomes, including posttraumatic growth, positive affect, and the integration of stressful life experiences, immediately following the training. These improvements were generally maintained until the follow-up assessment, 18 months later, indicating the potential for enduring positive effects. Additionally, participants showed significant reduction in symptoms related to PTSD, depression, anxiety, stress, insomnia, and negative affect. These reductions in symptom-related measures were also maintained until the follow-up assessment. Together, the evidence indicates that the employed posttraumatic growth-oriented training program provides benefits to veterans extending beyond increases in posttraumatic growth, addressing many of the issues that veterans face following exposure to trauma.

An important implication of the impact of this training program involves the use of peer-based delivery. The substantial impact of this peer-based approach suggests that a properly designed program delivered by peers who have a high degree of cultural competence may provide a unique pathway to addressing mental health needs. There is also the implication that peers may be especially effective at delivering such programs as they optimize the important relational component that is central to success.

## Data availability statement

The datasets presented in this article are not readily available because of concerns for confidentiality. Requests to access the datasets should be directed to the corresponding author, GE, gary_elkins@baylor.edu

## Ethics statement

The studies involving humans were approved by Baylor University’s Institutional Review Board. The studies were conducted in accordance with the local legislation and institutional requirements. The participants provided their written informed consent to participate in this study.

## Author contributions

JR: Conceptualization, Data curation, Formal analysis, Investigation, Methodology, Writing – original draft, Writing – review & editing. RT: Conceptualization, Data curation, Investigation, Methodology, Project administration, Supervision, Writing – review & editing. BM: Conceptualization, Data curation, Investigation, Methodology, Project administration, Supervision, Writing – review & editing. CA: Conceptualization, Writing – review & editing. GE: Conceptualization, Data curation, Formal analysis, Investigation, Methodology, Supervision, Writing – original draft, Writing – review & editing.
